# Association between *PPAP2B* gene polymorphisms and coronary heart disease susceptibility in Chinese Han males and females

**DOI:** 10.18632/oncotarget.14486

**Published:** 2017-01-04

**Authors:** Yu-Xiao Sun, Chuan-Yu Gao, Yang Lu, Xin Fu, Jun-Ge Jia, Yu-Jie Zhao, Lian-Dong Li, Hong-zhi Dui, Xing-Yu Zhang, Zhi-Ying Li, Lei Lei, Wei-Feng Zhang, Yi-Qiang Yuan

**Affiliations:** ^1^ Department of Cardiology, The Seventh People's Hospital, Zhengzhou, Henan, 450016, China; ^2^ Department of Cardiology, Henan Provincial People's Hospital, Zhengzhou, 450000, China; ^3^ Department of Medicine, Xi'an Jiaotong University, Xi'an, Shaanxi 710061, China; ^4^ Department of Cardiology, The First Affiliated Hospital of Zhengzhou University, Zhengzhou 450052, China

**Keywords:** coronary heart disease (CHD), single nucleotide polymorphisms (SNPs), PPAP2B, Chinese Han population, case-control study

## Abstract

Little is known about gender-related differences in the association between *PPAP2B* single nucleotide polymorphisms (SNPs) and coronary heart disease (CHD) in Chinese Han males and females. We therefore conducted a case-control study with 456 cases and 685 healthy controls divided into male and female subgroups. Five *PPAP2B* polymorphisms (SNPs) were selected and genotyped using Sequenom Mass-ARRAY technology. Odds ratios (OR) and 95% confidence intervals (CIs) were calculated using unconditional logistic regression adjusting for age and gender. Allelic model analysis revealed that for *PPAP2B* rs1759752, allele frequency distributions differed between cases and controls in the male subgroup (*p* = 0.015, OR: 1.401, 95%CI: 1.066–1.481). Genetic model analysis revealed that in the male subgroup, rs1759752 was associated with increased CHD risk in the dominant model (*p* = 0.035) and overdominant model (*p* = 0.045). In the female subgroup, rs12566304 was associated with a decreased CHD risk in the codominant model (*p* = 0.038) and overdominant model (*p* = 0.031). Additionally, the “GC” haplotypes of rs1759752 and rs1930760 were protective against CHD in males. These observations shed new light on gender-related differences in the association between *PPAP2B* gene polymorphisms and CHD susceptibility in the Chinese Han population.

## INTRODUCTION

Coronary heart disease (CHD) is a major cause of death and disability worldwide [1]. The incidence of CHD is higher in males than females. In China, moreover, the prevalence of CHD is increasing. One large study from the U.S. puts the annual mortality rate due to CHD there at 5% among males and 3.65% among females [2]. CHD is characterized by remodeling and narrowing of the coronary arteries, which supply blood, and thus oxygen, to the heart [3]. Twins study has shown that heritable factors account for 30-60% of inter-individual variation in the risk of CHD [4], which means genetic factors are key to an individual's susceptibility to CHD.

Various genomic regions and gene polymorphisms have been identified as being associated with the risk of CHD [5–7]. To date, genome-wide association studies (GWAS) have identified more than 40 common variants associated with the risk of CHD [8, 9]. For example, *PPAP2B* encodes lipid phosphate phosphatase 3 (LPP3), which is required for vascular development and may also contribute to the pathogenesis of human atherosclerotic disease [10]. A previous study showed that the rs17114046 single nucleotide polymorphism (SNP) in the final intron of *PPAP2B* is associated with an increased risk of CHD in Europeans and South Asians [11]. Another recent study observed that the CHD protective A allele of rs72664324 at the *PPAP2B* locus increases the transcriptional enhancer response to oxidized low-density lipoprotein in macrophages, which results in an alteration in LPP3 activity, and in turn promotes increased metabolism of pro-inflammatory mediators within atherosclerosis lesions [12].

The abovementioned studies suggest genetic polymorphisms in *PPAP2B* are closely associated with the risk of CHD. On the other hand, little is known about the contribution of *PPAP2B* SNPs to gender-related differences in CHD risk, especially in the Chinese Han population. We therefore performed a case-control study to investigate the associations between *PPAP2B* SNPs and the risk of CHD in Chinese Han males and females.

## RESULTS

We recruited 456 cases (291 males and 165 females; average age at diagnosis: 59.56, 64.01) and 685 controls (385 males and 300 females; average age: 47.55, 49.93) for this study. The characteristics of the cases and controls are shown in Table 1. We divided the samples into male and female subgroups, and multivariate analyses were adjusted for age.

**Table 1:** Basic characteristics of CHD cases and healthy controls

Variables	Male			Female		
	Case	Control	*p*	Case	Control	*p*
Number	291	385		165	300	
Age yr (mean ± sd)	59.56±12.18	47.55±10.655	< 0.001	64.01±10.74	49.93±7.743	< 0.001
ALB (g/L)	40.86±4.50			41.13±0.30		
ALB/GLOB	1.66±0.26			1.59±0.01		
ALT (U/L)	32.32±34.17			29.09±4.61		
AST (U/L)	38.16±69.46			33.32±3.49		
AST/ALT	1.23±0.94			1.42±0.08		
aPOA1 (g/L)	1.22±0.24			1.33±0.02		
aPOB (g/L)	0.97±0.37			1.05±0.03		
aPOA1/aPOB	1.45±0.71			1.47±0.06		
Ca(mmol/l)	2.29±0.16			2.29±0.01		
GGT (U/L)	47.06±69.30			40.39±7.46		
GLU (mmol/L)	6.27±2.23			6.45±0.21		
HDL-C (mmol/L)	1.10±0.25			1.19±0.02		
LDL-C (mmol/L)	1.86±0.89			2.02±0.57		
Lp(a) (mg/L)	227.16±253.31			260.70±19.93		
MPV (fl)	13.27±7.29			12.75±0.59		
PLT (109/L)	163.19±72.13			180.38±0.29		
PCT (%)	1.25±3.45			0.94±0.22		
PDW (%)	14.32±2.79			14.07±0.26		
TP(g/L)	69.51±6.63			67.229±0.455		
TG (mmol/L)	1.80±1.65			1.80±0.10		
TC (mmol/L)	3.96±1.19			4.33±0.09		
UA(umol/l)	311.77±85.46			265.80±6.24		

ALB: albumin, ALB/GLOB: albumin/globulin, ALT: alanine aminotransferase, AST: aspartate aminotransferase, apoA: apolipoprotein A, apoB: apolipoprotein B, GGT: gamma-glutamyl transpeptidase, GLU: glucose, HDL: high-density lipoprotein, LDL: low-density lipoprotein, LP(a): lipoprotein, MPV: Mean Platelet Volume, PLT: platelet, PCT: plateletcrit, PDW: platelet distribution width, TP: total protein, TG: triglyceride, TC: total cholesterol, UA: Uric acid.

*p* values were calculated using the Welch's test; *p* < 0.001 indicates statistical significance.

A total of five *PPAP2B* SNPs were identified in the cases and controls. All SNP call rates exceeded 98.2%, which was considered high enough to perform association analyses. In the controls, all SNPs were in Hardy-Weinberg equilibrium (HWE) (Table 2). For SNP rs1759752, the allele frequency distributions differed between male cases and controls. (*p* = 0.015, OR: 1.401, 95%CI: 1.066-1.481), whereas there was no significant difference in the female subgroup. We also compared the genotype frequencies between cases and controls. For SNP rs1759752, the genotype frequency distributions also differed between the male cases and controls (*p* = 0.019), while for rs12566304, the genotype frequency distributions differed between female cases and controls (*p* = 0.020) (Table 3).

**Table 2:** Allele frequencies in cases and controls and odds ratio estimates for CHD

SNP ID	Gene(s)	Band	Alleles A^a^/B	MAF		HWE*p*	ORs	95% CI	*p*
				Case	Control				
Male									
rs11206831	PPAP2B	1p32.2	T/C	0.050	0.055	1	0.909	0.559-1.478	0.700
rs1759752	PPAP2B	1p32.2	C/G	0.220	0.168	0.8558	1.401	1.066-1.841	0.015*
rs1930760	PPAP2B	1p32.2	T/C	0.490	0.467	0.4733	1.093	0.881-1.356	0.418
rs12566304	PPAP2B	1p32.2	A/C	0.132	0.148	0.1561	0.877	0.643-1.198	0.410
rs914830	PPAP2B	1p32.2	T/C	0.324	0.329	0.8172	0.976	0.776-1.229	0.838
Female									
rs11206831	PPAP2B	1p32.2	T/C	0.050	0.045	1	1.011	0.530-1.928	0.975
rs1759752	PPAP2B	1p32.2	C/G	0.209	0.165	1	1.338	0.950-1.883	0.094
rs1930760	PPAP2B	1p32.2	T/C	0.442	0.498	0.7299	0.799	0.610-1.046	0.102
rs12566304	PPAP2B	1p32.2	A/C	0.161	0.148	0.1653	1.099	0.759-1.591	0.619
rs914830	PPAP2B	1p32.2	T/C	0.339	0.348	0.1621	0.961	0.724-1.246	0.784

MAF, minor allelic frequency; HWE, Hardy-Weinberg Equilibrium; ORs, odds ratios; CI: confidence interval.

^a^ Minor allele; *p ≤ 0.05 indicates statistical significance;

**Table 3:** Genotype distribution for SNPs in CHD patients and healthy controls

SNP ID	Alleles A^a^/B	Genotype	Male (No.)		*p*^b^	Female (No.)		*p*^b^
			Case	Control		Case	Control	
rs11206831	T/C	CC	262	344	0.942	150	273	1
		TC	29	40		15	27	
		TT	0	1		0	0	
rs1759752	C/G	GG	172	205	0.019*	103	209	0.246
		CG	110	106		55	83	
		CC	9	11		7	8	
rs1930760	T/C	CC	76	105	0.607	55	77	0.208
		TC	145	199		74	147	
		TT	70	80		36	76	
rs12566304	A/C	CC	223	283	0.624	120	214	0.020*
		AC	59	90		37	83	
		AA	9	12		8	3	
rs914830	T/C	CC	130	174	0.765	69	133	0.240
		TC	132	167		80	125	
		TT	28	43		16	42	

^a^ Minor alleles, OR: Odds ratio, CI: Confidence interval.

^b^: *p* values were calculated from Pearson's chi-square test adjusted for age.

* *p* ≤ 0.05 indicates statistical significance.

We assumed the minor allele of each SNP was a CHD risk factor compared to the wild-type allele and analyzed associations between SNPs and CHD in various inheritance models (Table 4). In the male subgroup, rs1759752 was associated with an increased CHD risk in the dominant model (*p* = 0.035, OR: 1.47, 95%CI: 1.03-2.11) and overdominant model (*p* = 0.045, OR: 1.46, 95%CI: 1.01-2.11). In the female subgroup, rs12566304 was associated with a decreased CHD risk in the codominant model (*p* = 0.038, OR: 0.57, 95%CI: 0.32-1.00) and overdominant model (*p* = 0.031, OR: 0.54, 95%CI: 0.31-0.96).

**Table 4:** Association between significant SNPs and CHD in multiple inheritance models (adjusted for age)

SNP	Model	Genotype	Group=case	Group=control	OR (95% CI)	*p*	AIC	BIC
rs1759752in Male	Codominant	G/G	172 (59.1%)	265 (69.4%)	1	0.11	760.6	778.6
		G/C	110 (37.8%)	106 (27.8%)	1.48 (1.02-2.15)			
		C/C	9 (3.1%)	11 (2.9%)	1.40 (0.51-3.79)			
	Dominant	G/G	172 (59.1%)	265 (69.4%)	1	0.035*	758.6	772.2
		G/C-C/C	119 (40.9%)	117 (30.6%)	1.47 (1.03-2.11)			
	Recessive	G/G-G/C	282 (96.9%)	371 (97.1%)	1	0.69	762.9	776.4
		C/C	9 (3.1%)	11 (2.9%)	1.22 (0.45-3.30)			
	Overdominant	G/G-C/C	181 (62.2%)	276 (72.2%)	1	0.045*	759	772.6
		G/C	110 (37.8%)	106 (27.8%)	1.46 (1.01-2.11)			
	Log-additive	—	—	—	1.37 (1.00-1.88)	0.049	759.2	772.7
rs12566304in Female	Codominant	C/C	120 (72.7%)	214 (71.3%)	1	0.038*	411.9	428.5
		C/A	37 (22.4%)	83 (27.7%)	0.57 (0.32-1.00)			
		A/A	8 (4.8%)	3 (1%)	2.83 (0.60-13.46)			
	Dominant	C/C	120 (72.7%)	214 (71.3%)	1	0.14	414.2	426.6
		C/A-A/A	45 (27.3%)	86 (28.7%)	0.67 (0.39-1.15)			
	Recessive	C/C-C/A	157 (95.2%)	297 (99%)	1	0.11	413.9	426.3
		A/A	8 (4.8%)	3 (1%)	3.32 (0.71-15.53)			
	Overdominant	C/C-A/A	128 (77.6%)	217 (72.3%)	1	0.031*	411.8	424.2
		C/A	37 (22.4%)	83 (27.7%)	0.54 (0.31-0.96)			
	Log-additive	—	—	—	0.84 (0.53-1.33)	0.45	415.8	428.3

ORs, odds ratios; CI: confidence interval; AIC: Akaike's Information criterion; BIC: Bayesian Information criterion.

* *p* ≤0.05 indicates statistical significance.

The basic characteristics of the study subjects stratified by genotype are shown in Table 5. In the male subgroup, we found significant differences in the albumin (ALB), apolipoprotein A (aPOA1) and total protein (TP) concentrations, as well as the alanine aminotransferase (ALT)/aspartate aminotransferase (AST) ratio among differnet *PPAP2B* rs1759752 genotypes (*p* < 0.05). Subjects carrying the rs1759752 “CC” genotype had lower ALB, aPOA1 and TP concentrations and higher AST/ALT ratios than those carrying the “CG” and “GG” genotypes. In the female subgroup, we observed a significant difference in gamma-glutamyl transpeptidase (GGT) concentrations among the *PPAP2B* rs12566304 genotypes (*p* < 0.05). Patients who carried the minor C allele displayed lower GGT levels than those with the wild-type A allele.

**Table 5:** Clinical and biochemical characteristics of participants, stratified based on *PPAP2B* gene polymorphism

SNP	Characteristics	Genotypes (mean ± sd)			*p*
rs1759752in Male		CC	CG	GG	
	ALB	37.14±5.63	40.56±4.52	41.26±4.35	0.019
	AST/ALT	1.87±1.77	1.37±1.20	1.11±0.63	0.011
	aPOA1	1.02±0.16	1.20±0.22	1.24±0.25	0.014
	TP	60.22±8.33	65.45±6.58	66.53±6.42	0.010
rs12566304in Female		CC	AC	AA	
	GGT	34.21±62.98	33.83±36.38	159.63±335.72	0.001

ALB: albumin, ALB/GLOB: albumin/globulin, ALT: alanine aminotransferase, AST: aspartate aminotransferase, apoA: apolipoprotein A, GGT: gamma-glutamyl transpeptidase, HDL: high-density lipoprotein, TP: total protein, UA: Uric acid.

*p* ≤0.05 indicates statistical significance.

**Figure 1:**
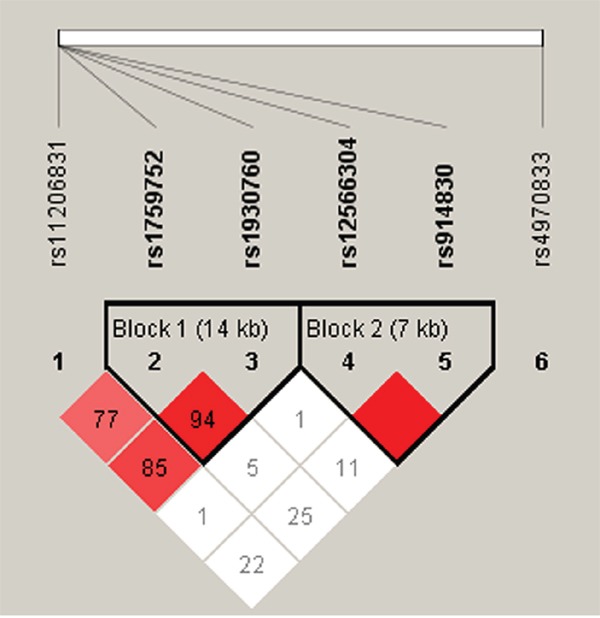
Haplotype block map for all the SNPs of the *PPAP2B* gene

In the male subgroup, haplotype analysis revealed two blocks in *PPAP2B* (Figure 1): block 1 included rs1759752 and rs1930760, while block 2 included rs12566304 and rs914830. Further analyses of associations between *PPAP2B* haplotypes and CHD risk showed that haplotype “GC” in block 1 was protective against CHD in males (*p* = 0.03, OR: 0.76, 95%CI: 0.59 - 0.97) (Table 6).

**Table 6:** *PPAP2B* haplotype frequencies and the association with the risk of CHD in male case and control patients (adjusted for age)

Block	SNP	Haplotypes	Freq-case	Freq-control	OR (95% CI)	*p*
1	rs1759752|rs1930760	GT	0.484	0.464	1	—
		GC	0.296	0.369	0.76 (0.59 - 0.97)	0.03*
		CC	0.215	0.164	1.28 (0.95 - 1.74)	0.11

* *p* ≤0.05 indicates statistical significance.

## DISCUSSION

In the present case-control study, we investigated the association between five *PPAP2B* SNPs and the risk of CHD in the Chinese Han population. We found that SNP rs1759752 is associated with an increased risk of CHD in males, while rs12566304 is associated with a decreased risk of CHD in females. Additionally, we observed that haplotype “GC” of rs1759752 and rs1930760 was protective against CHD in males.

CHD was once considered as a cholesterol storage disease, but is now known to reflect complex interactions among molecular messages within the cells of the artery wall and in the blood [13]. Mammals express three lipid phosphate phosphatases (LPPs): LPP1, LPP2 and LPP3 encoded by *PPAP2A*, *PPAP2C* and *PPAP2B*, respectively. In mice, loss of *PPAP2A* [14] or inactivation of *PPAP2C* [15] does not result in phenotypic alteration. However, *PPAP2B* knockout is embryonically lethal, mainly due to defects in extra-embryonic vascular development [10], which suggests *PPAP2B* plays an important role in vascular development.

*PPAP2B* is located at locus 1p32.2. It is abundantly expressed in human plaque foam cells, and its specific enzymatic activity in macrophages was increased by oxidized low-density lipoprotein [12]. In our study, we found that males who carried the rs1759752 “CC” genotype had lower ALB, aPOA1and TP concentrations and a higher AST/ALT ratio than those carrying the “CG” or “GG” genotype. In addition, female patients who carried the minor C allele of rs12566304 displayed lower GGT levels than those carrying the wild-type A allele. It is noteworthy, that lower ALB, aPOA1, TP and GGT levels, which may reflect increased proteinase activity, and higher AST/ALT ratios are always associated with both liver and cardiovascular disease. We therefore suggest that *PPAP2B* rs1759752 and rs12566304 SNPs may affect CHD risk by influencing LPP3 enzymatic activity.

Earlier GWAS studies revealed several common variants that influence CHD risk in populations of European ancestry. These include rs1333049, rs599839, rs3008621, rs501120, rs6922269 and rs2943634, among others [16]. Chinese researchers have replicated these studies to explore novel loci in the Chinese population, including rs6903956, rs2123536, rs1842896, rs9268402 and rs7136259 [17–19]. In the present study, we showed that rs1759752 is associated with increased CHD risk in males, while rs12566304 is associated with decreased CHD risk in females.

The present study has several potential limitations. First, the sample size is relatively small, and the participants included only Chinese population. Second, CHD is a very heterogeneous disease with many traditional risk factors, including tobacco use, physical inactivity, poor nutrition, obesity, hypertension, high blood cholesterol, and diabetes. We could not completely eliminate the potential influences of these factors on the results.

In sum, our results indicate that *PPAP2B* rs1759752 is associated with an increased risk of CHD in males, while rs12566304 was associated with a decreased risk of CHD in females. These SNPs may have the potentially to serve as prognostic biomarkers for CHD among the Chinese Han population. Further study will focus on determining the functional role of these SNPs and on validating our findings with a larger sample.

## MATERIALS AND METHODS

### Ethics statement

We strictly obeyed the World Medical Association Declaration of Helsinki when using human tissue and discussing the study protocol with subjects. The protocol was approved by the Ethical Committee of the Seventh People's Hospital of Zhengzhou. Each participant provided written, informed consent.

### Subjects

All participants in our study were Han Chinese lived in Henan Province. A total of 456 CHD patients and 685 healthy controls were consecutively recruited between May 2013 and July 2015 in the Seventh People's Hospital of Zhengzhou, China. Patients were diagnosed with CHD using standard coronary angiography, which revealed ≥ 70% stenosis of the main branch of a coronary artery or aortic stenosis ≥ 50%. Subjects with myocardial infarction, stable angina and unstable angina were classified as CHD subjects. There were no age, sex or disease-stage classification restrictions when enrolling the case group. Excluded were subjects with chronic diseases or conditions involving the brain, liver, heart or lung, and patients with more advanced cardiovascular, metabolic or endocrine diseases. Controls were healthy people receiving physical examinations in other clinical departments of Tangdu Hospital. Healthy controls did not have congenital heart disease, familial hypercholesterolemia, end-stage renal disease or vasculitis, which could have affected our study results. Peripheral blood was collected from both cases and controls for DNA extraction.

### Clinical data and patient demographics

A standardized epidemiological questionnaire was provided to all subjects to collect basic demographic information, including sex, age, residence, educational status, history of family cancer, history of smoking and alcohol consumption. Plasma carcinoembryonic antigen and alpha-fetoprotein levels were determined to ensure that no controls suffered from any cancer.

### SNP selection and genotyping

Candidate *PPAP2B* SNPs were selected from previous publications that associated polymorphisms with CHD [20, 21]. SNPs with minor allele frequencies (MAF) > 5% in the HapMap CHB population were selected. We validated five SNPs in *PPAP2B*. A GoldMag-Mini Purification Kit (GoldMag Co. Ltd. Xian city, China) was used to extract genomic DNA from whole blood samples. DNA concentrations were measured using a DU530 UV/VIS spectrophotometer (Beckman Instruments, Fullerton, CA, USA). Using MassARRAY Assay Design 3.0 software (Sequenom, San Diego, CA, USA), we designed a multiplexed SNP MassEXTENDED assay [22]. SNPs were genotyped using the standard protocol recommended by the MassARRAY RS1000 (Sequenom) manufacturer, and data were analyzed using Typer 4.0 Software (Sequenom). The primers used for this study were listed in Table 7.

**Table 7:** Primers used for this study

SNP_ID	1st_PCRP	2st_PCRP	UEP_SEQ
rs11206831	ACGTTGGATGTGACCTCACTGAGTCTGAAC	ACGTTGGATGAGGCAGGAACTATGCCTGTG	ccTCCTCACACCTTTGCAAGT
rs1759752	ACGTTGGATGTCAGGGAGTCGACAACACAG	ACGTTGGATGGGGTTAGGATTACAAGAAGC	tagtATTACAAGAAGCCAAGTCCG
rs1930760	ACGTTGGATGGCACTGGCTCAAGTTAACTG	ACGTTGGATGTTTAAACTCCAGGCTATTGC	gaggTTGATGAAATTAGTTACCTAGC
rs12566304	ACGTTGGATGGAATGTTGGCACTGACAATG	ACGTTGGATGTCTCCTTATTCCCATGTGAC	ggttaATGTGACTGAGGATGAAACA
rs914830	ACGTTGGATGGGAGACGACTTTTACTGAGC	ACGTTGGATGTCTTGGCCTGTACTTGAGTG	gTGAGTGGGTCTGGGCGT

UEP: Unextended mini-sequencing primer.

### Statistical analysis

We used the Microsoft Excel and SPSS 17.0 (SPSS, Chicago, IL) statistical packages to perform the statistical analyses. All *p* values were two-sided, and *p* < 0.05 was considered statistically significant. A *t* test and Chi-squared test were performed to compare sex and age differences between cases and controls. Fisher's exact test was applied to each SNP in the controls to test for departure from the Hardy-Weinberg Equilibrium (HWE). Odds ratios (ORs) and 95% confidence intervals (CIs) for the allele and genotype frequencies were calculated using the Pearson Chi-square test adjusted for age (sex subgroups were separated) [23]. The Cochran-Armitage trend test was used to determine associations between SNPs and CHD.

PLINK software (http://pngu.mgh.harvard.edu/purcell/plink/) was used to assess SNP associations with CHD risk in different genetic models (Codominant, dominant, recessive, overdominat and additive). We used unconditional logistic regression analysis to calculate ORs and 95% CIs adjusted for age [24]. Pairwise linkage disequilibrium and haplotype constructions were performed using HAPLOVIEW 4.1 (http://broad.mit.edu/mpg/haploview) [25].
